# Microvascular pathology in the spinal cord of severe spinal muscular atrophy patients

**DOI:** 10.21203/rs.3.rs-8085055/v1

**Published:** 2025-12-01

**Authors:** Hazel Allardyce, Heather Lanz, Benjamin D. Lawrence, Thomas O. Crawford, Charlotte J. Sumner, Simon H. Parson

**Affiliations:** University of Aberdeen; University of Aberdeen; University of Aberdeen; Johns Hopkins University School of Medicine; Johns Hopkins University School of Medicine; University of Aberdeen

**Keywords:** Von Willebrand factor, Endothelial cell, BSCB, Astrocytes, Pericytes, Microglia

## Abstract

Severe spinal muscular atrophy (SMA) is a life-limiting neurodegenerative disease of infancy and early childhood, caused by reduced expression of the ubiquitous survival motor neuron protein (SMN). While current therapies aim to increase SMN levels and preserve motor neurons, significant deficits remain in treated patients and non-neuronal manifestations of SMN deficiency are underexplored. Vascular abnormalities including intrinsic endothelial cell dysfunction, altered vessel morphology, and altered vascular distribution have been reported in preclinical SMA models. Here, we characterised vascular architecture and blood-spinal cord barrier (BSCB) morphology and integrity in post-mortem spinal cord samples from severe SMA patients compared with unaffected controls. Von Willebrand Factor (VWF), a marker of endothelial cell function, was reduced within individual vascular endothelial cells, and associated with ultrastructural endothelial cell oedema, vacuolisation and compromised endothelial integrity. Ultrastructural damage extended to other components of the blood-spinal cord barrier (BSCB) as evidenced by extravascular leakage of fibrinogen into the neural parenchyma and microglial activation consistent with a neuroinflammatory environment. Together, these findings suggest that vascular defects with associated dysfunction of the BSCB are present in the spinal cord of infants with severe SMA. This work adds to a growing body of evidence linking microvascular dysfunction to neurodegeneration in human neurodegenerative diseases. Further studies are warranted to define the contribution of vascular dysfunction to SMA pathogenesis and to assess whether current therapies adequately address this aspect of the disease.

## Introduction

Severe spinal muscular atrophy (SMA) is a genetic neuromuscular disorder of infancy and early childhood caused by reduced levels of the survival motor neuron (SMN) protein. SMA primarily manifests pathologically as progressive motor neuron loss, and clinically with skeletal muscle weakness and denervation-induced atrophy [44]. These hallmark features are amenable, at least in part, to treatment with any of three SMN-enhancing therapies, nusinersen (Spinraza; Biogen), risdiplam (Evrysdi; Roche), or onasemnogene abeparvovec (Zolgensma; AveXis, Novartis). Commensurate with the amount of neurodegeneration present before treatment is given, the clinical course and prognosis of SMA is greatly improved [15].

While classic clinical and pathologic studies of SMA have reasonably focused on impaired motor neurons and skeletal muscle, a wider but clinically more subtle dysfunction is suggested by the ubiquitous expression of SMN. Studies in animal models support this, revealing broad, multi-system pathology [31]. Because the extent and distribution of these features differs widely between animal models, and the SMN-expression threshold below which tissue and species’ dysfunction manifests is unknown, the relevance of these extra-neuronal features to humans with SMA is uncertain. With improved survival following central nervous system (CNS)-targeted therapies, there is reason for concern that previously underappreciated, non-motor manifestations of SMA may emerge.

Our focus on the vasculature stems from both clinical and preclinical evidence that suggests vascular endothelium may be another tissue relatively vulnerable to diminished SMN expression. Retinal imaging studies in older children with milder forms of SMA demonstrate reduced capillary branching complexity, a finding mirrored in pathological studies of SMA mouse models [73]. Circulating biomarkers further support the presence of vascular damage, as SMA patients exhibit elevated circulating endothelial cells (CEC) and reduced endothelial progenitors, which correlate with both disease severity and SMN2 copy number [14, 19, 73]. A small number of SMA patients have presented with distal digital necrosis, mirroring tail and ear necrosis in SMA mouse models [6, 58]. Finally, congenital heart defects are more commonly observed in infants with the most severe forms of SMA [57] which are potentially linked to early endothelial dysfunction, as vascular endothelial precursors are essential for heart development [27, 72].

Beyond these clinical indications, pathological findings in both human and animal SMA tissue also reveal a range of vascular abnormalities. Skeletal muscle biopsies from SMA patients across the severity spectrum show reduced vascularisation and disrupted vessel architecture, marked by narrow vessels and simplified capillary beds [63]. Similar vascular defects are reported in systemic organs of SMA models, including the heart, kidneys, and intestines [3, 41, 61, 62]. These vascular abnormalities may arise from intrinsic endothelial defects, as endothelial cells with depleted SMN exhibit impaired angiogenic behaviours, including reduced migration and tube formation, shown in SMN-deficient human umbilical vein endothelial cells (HUVECs) cultures and aortic endothelial cells from severe SMA mouse models [73].

Importantly, vascular pathology may also contribute directly to CNS degeneration. In severe SMA mouse models, loss of the endothelial marker platelet endothelial adhesion molecule 1 (PECAM-1) in spinal cord grey and white matter preceded the onset of motor symptoms, progressed with age and was accompanied by pathological evidence of blood spinal cord barrier (BSCB) changes and tissue hypoxia, supported by increased uptake of in-vivo biomarkers of hypoxia identified pathologically [29, 63]. The spinal vasculature not only sustains neuronal and non-neuronal metabolism but also forms the structural core of the BSCB, regulating immune cell and molecular entry into the CNS [2]. Therefore, endothelial dysfunction within the spinal cord has the potential to alter the neural microenvironment and exacerbate neurodegeneration [21]. Indeed, vascular changes are increasingly recognised across other neurodegenerative diseases such as amyotrophic lateral sclerosis, Alzheimer’s disease, and Parkinson’s disease, though it remains debated whether these represent primary drivers of pathology or secondary consequences of neurodegeneration [12, 24, 49, 64, 71].

These observations suggest that vascular endothelial dysfunction may contribute both to local spinal cord pathology and to wider systemic involvement in SMA. This highlights the need for focused investigation of the human vascular network in SMA. In this study, we characterised the spinal cord vasculature and BSCB in autopsy tissue from a cohort of infants with well-defined, severe SMA and previously quantified motor neuron loss [4]. Our analysis reveals aberrant vascular architecture and BSCB leakage, implicating vascular dysfunction as a potential contributor to disease progression. While centred on the spinal cord, these findings also provide testable hypotheses for extra-neuronal SMA pathology.

## Methods

### Sample collection

Expedited autopsies were conducted under parental- or patient-informed consent in strict observance of the legal and institutional ethical regulations, and material tissue agreements were obtained between collaborating institutes. Paediatric human thoracic spinal cord samples from SMA patients (n=6) and similarly age-matched controls (n=6) were obtained from brain banks: BRAINUK, NIH Neurobiobank, and John Hopkins University Medical School. Tissue samples were dissected at autopsy, fixed in 4% paraformaldehyde at room temperature for 24 hours, washed and stored in PBS. Samples prepared for transmission electron microscopy from SMA patients (n=4), and similarly age-matched controls (n=2) were dissected and fixed in 2% glutaraldehyde at 4°C for 24 hours. Patient demographics of samples is detailed in Table 1. The following analyses were conducted blinded to disease status (SMA or control).

### Immunofluorescence/immunohistochemistry

Histology samples were cryopreserved in 30% sucrose solution with 0.1% sodium azide and embedded in optimum cutting temperature compound. Serial, transverse spinal cord sections were prepared (8μm). Sections were air dried overnight and submerged in 10mM sodium citrate buffer, pH6 (20 mins at 95°C) for antigen retrieval, cooled in solution and washed (5 mins) in 0.1M PBS. Autofluorescence was quenched using 0.1% Sudan black (20 mins) and sections were washed three times (2× 10 mins PBST (0.1M PBS with 0.1% Tween-20), 1× 0.1M PBS). Sections were incubated (2 hours at 4°C) in blocking solution (0.4% bovine serum albumin (BSA), 1% Triton X-100 in 0.1M phosphate buffered saline (PBS), 5% donkey serum), and then with diluted primary antibody; anti-Von Willebrand Factor 1:100 (Abcam, ab9378), anti-glial fibrillary acidic protein (GFAP) 1:100 (Abcam, ab53554), anti-haemoglobin 1:200 (R&D Systems, G-134-C), anti-fibrinogen 1:1000 (Millipore, 341552), anti-Iba1 1:500 (Abcam, ab107159), anti-CD68 1:200 (Abcam, ab213363) (overnight at 4°Cc). Sections were washed three times as before (2× 0.1M PBST, 1× 0.1M PBS), then incubated with corresponding diluted secondary antibody; donkey anti-rabbit AF+555 (Invitrogen, A32794), donkey anti-rabbit AF488 (Invitrogen, A21206), donkey anti-goat 488 (Abcam, ab150129), donkey anti-goat Cy3 (Abcam, ab6949) at 1:200 (2 hours at 4°C) with successive washes as before. For double staining, the protocol was repeated sequentially. Nuclei were counterstained using DAPI (4′,6-diamidino-2-phenylindole) at 1:20,000 (10 mins at room temperature), then washed as before. Sections were mounted using MOWIOL media (10% Mowiol (Sigma-Alrich, 81 381), 20% glycerol, 50% 0.2M Tris buffer pH 8.5, 3% 1,4-diazobicyclooctance in distilled water). Negative control slides which lacked primary antibody were included in each staining run for detection of autofluorescence and non-specific binding.

Immunohistochemistry used the Novolink Polymer Detection kit (Lecia Biosystems, RE7280-K) and DAB visualisation for primary antibodies anti-Ki67, a proliferation marker [11, 25], 1:50 (Abcam, ab16667) and anti-fibrinogen 1:2000 (Millipore, 341552) following the supplied instructions. Sections were coverslipped with DPX mounting media. All stained sections were imaged on an EVOS M5000 inverted microscope.

### VWF Quantification

Systematic uniform random sampling was implemented using a random number generator to determine the first section in the series and every 12^th^ section of spinal cord was immunofluorescently stained with VWF. Sections were imaged at 10x magnification in the grey matter ventral and dorsal horns, and white matter corticospinal tract and dorsal column. Micrographs were analysed using FIJI (Image J) software. For each slide, threshold was set manually to the point of saturation and the de-speckle function was applied to subtract small background noise. Automated thresholding was not applicable due to differences in background fluorescence. A counting frame test probe composed of 12 counting boxes of 17578μm^2^ area was overlain on brightfield micrographs and employed as described in [20], each individual counting box representing a field of view (FOV). FOVs that extended beyond the region of interest were identified and discounted from analysis. Sudan black counterstaining allowed delineation of white and grey matter under brightfield illumination.

#### VWF+ Area/Field of View:

For each included FOV, total VWF stained area was calculated relative to total field of view (area of counting box) and expressed as % area stained. FOV analysed, Control > 150 and SMA >150 per region of interest.

#### Vessel density:

Vessel density/per mm^2^ was calculated for each ROI on each slide by dividing the total number of vessel profiles by the total area of FOV included (17578μm^2^ *X* number of grid boxes analysed) and converted to vessels/mm^2^. Mean vessel density, in each individual ROI across all sections analysed, was calculated. FOV analysed, Control > 150 and SMA >150 per region of interest. Simple linear regression analyses show change in spinal cord vascular density between ages.

#### VWF+ Area/vessel profile:

Individual VWF^+^ vessel profiles (200 per spinal cord sample) were assessed within the ventral horn. Individual vessel profiles were manually selected and stained area was measured using particle analysis on FIJI (ImageJ), providing the stained area per vessel profile.

### Transmission electron microscopy

Epoxy resin-embedded blocks for transmission electron microscopy (TEM) were prepared at Johns Hopkins University (Control n=2, SMA n=4), as described in [35]. Grids were sectioned by the microscopy and histology facility at the University of Aberdeen using a diamond knife to cut approximately 90 nm sections onto formvar/carbon-coated copper grids. The sections were then contrast-stained with Uranyless (Labtech, 11000-200) and Lead citrate. Grids were surveyed systematically: tissue was randomly oriented, and imaging began at the top left corner of a randomly oriented section, proceeding from left to right on each row, and then moving to the row below in a right-to-left direction. The first 50 vessel profiles identified in grey matter were imaged from one section per case. Grey matter was identified by the surrounding neuropil and the absence of myelinated axons around the vessel. Vessel profiles that overlay grids were excluded, thus constraining analysis to vessels less than 30 μm diameter and biasing to smaller vessels in that pool.

#### Endothelial cell area:

The luminal and basal boundaries of the endothelial cells were traced, and the area of the lumen was subtracted from the area enclosed by the outer basal boundary to calculate the endothelial cell area.

#### Endothelial cell frequency:

Determined by calculating the frequency of endothelial cell nuclei observed in vessel images.

#### Minimum lumen diameter:

To determine minimum lumen diameter, electron micrographs were assessed using FIJI (ImageJ) software. Minimum lumen diameter was chosen to reflect the minimum vessel width irrespective of plane of section. Lumenal boundaries of endothelial cells were manually traced, and an ellipse was fitted to the structure to allow maximum and minimum lumen measurements through the centroid.

#### Morphological EM observations:

All observations of morphological differences in endothelial cells, basement membrane, pericytes and astrocytes were identified using published literature as reference [24, 36, 45]. Phenotype frequency determined as a percentage of total vessels assessed.

### Extravascular leakage

#### Fibrinogen/Haemoglobin:

Three sections of spinal cord, selected using a random number generator, from control (n=5) and SMA (n=5) tissues were immunohistochemically stained for fibrinogen, VWF and haemoglobin as described above. Extravascular fibrinogen/haemoglobin was determined when staining was observed outside the walls of a vessel in either the grey or white matter of the spinal cord and there was a visible vessel within close proximity/surrounded by positive staining. Extravascular fibrinogen was defined by either a diffuse pattern of fibrinogen positive staining surrounding a vessel, or a small, clustered deposit of fibrinogen positive staining in the perivascular space. Extravascular haemoglobin was defined by a region of haemoglobin positive staining either entirely or partially surrounding a vessel. Number of vessels exhibiting extravascular leakage of fibrinogen and haemoglobin was quantified by counting all visible vessel profiles in a sections, and expressing the number of vessels associated with extravascular fibrinogen of haemoglobin as a percentage of the total.

### Microglial Abundance and activation

#### Iba1/CD68:

Three sections of spinal cord, selected using a random number generator, from control (n=4) and SMA (n=4) were immunofluorescently stained for Iba1 (microglial marker) and CD68 (marker of microglial activation) and DAPI. Microglial cell bodies (nuclear DAPI with surrounding Iba1 positive staining) were counted to determine microglial abundance. Microglial activation was determined through co-localised Iba1 positive and CD68 positive staining. Of the total microglial (Iba1 positive) cells observed, the number of activated microglial (Iba1 positive and CD68 positive) cells are presented as a percentage.

### Statistical Analysis

Outliers were identified using ROUT method, Q=1%, and removed from the dataset prior to analysis. Descriptive statistics and D’Agostino & Pearson normality tests were conducted to determine distribution of data. If data was of a Gaussian distribution, data is presented as mean +SEM, however if of a non-Gaussian distribution, results are presented as median (interquartile range). Statistical comparison was analysed by Welch’s T-tests. Under all circumstances, *P < 0.05, **P < 0.01 and ***P < 0.001.

## Results

### Patient population

We systematically assessed the spinal cord vasculature and blood–spinal cord barrier integrity in a cohort of infants diagnosed with severe spinal muscular atrophy, and with survival times ranging from 1 to 12 months.

Patient demographics are detailed in Table 1, and information concerning motor neuron loss and spinal cord development and growth for these cases are previously published [4]. All but one SMA case within this cohort, whose genotype remains unknown, possessed two copies of *SMN2.* This case has the shortest postnatal survival of just 7 days and is likely to reflect either 1 or 2 copies of *SMN2*, as 3 copies of the *SMN2* gene are associated with significantly longer survival [14, 43, 70].

As is inherent in all autopsy-based comparative pathology studies, differences in the cause of death and preterminal health status must be acknowledged. SMA cases died from SMA-related complications that involved extended periods of metabolic distress and prolonged respiratory decline, while controls died from sudden unexplained death syndrome (n=2), pneumonia (n=2), asphyxia (n=1), or unknown causes in three cases—mostly acute events that likely involve less prolonged systemic compromise than SMA cases. Postmortem intervals ranged from 7–32 hours in SMA cases and 14–69 hours in controls, with unknown intervals for two SMA cases and one control.

### VWF expression is decreased in endothelial cells in the SMA spinal cord

Spinal cord vasculature was visualized by immunostaining sections for von Willebrand factor (VWF), a widely used cytosolic marker of endothelial cells and endothelial cell health [59].

**Figure 1:**
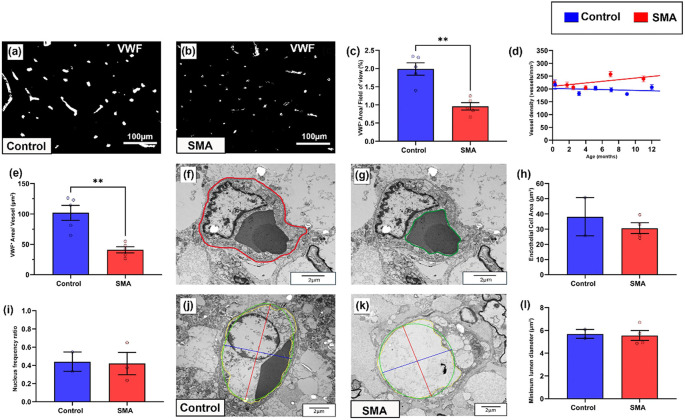
VWF expression is decreased in endothelial cells in the SMA spinal cord Representative micrographs of (a) control and (b) SMA ventral horn of the spinal cord labelled with Von Willebrand Factor (VWF) to visualise endothelial cells, scale 100μm. (c) Total VWF^+^ staining per FOV in control and SMA ventral horn (Welch’s T test, **P <0.005), mean ±SEM. Control n=5, SMA n=5. (d) Mean vascular density (vessels/mm^2^) in ventral horn ±SEM, with linear regression, 95% CI. Control n=6, SMA n=6, (e) Case average VWF positive staining per individual vessel in control and SMA ventral horn (Welch’s T test, **P <0.005), mean ±SEM. Control n=5, SMA n=5. (f-g) Electron micrograph of endothelial cell with annotated outer boundary - red (f) and inner boundary - green (g). (h) Endothelial cell area, mean ±SEM. (i) Frequency ratio of endothelial cell nuclei, mean ±SEM. Representative electron micrographs of (l) control and (m) SMA vessels showing luminal diameter measure, scale 2μm. (j) Minimum luminal diameter in control and SMA grey matter, mean ±SEM. Control n = 2, SMA n = 4.

As the primary site of neurodegeneration in SMA, we first analysed the ventral horn. In control cases, VWF staining of individual blood vessels was bright and uniform in contrast to SMA cases, where vessels were more heterogeneously stained (Fig. 1a–b). Total VWF positive stained area (expressed as a percentage of total area stained) was significantly lower in SMA ventral horn compared to age-matched controls: control = 1.99% (±0.17), and SMA = 0.79% (±0.10), mean (±SEM), **P <0.01 (Fig. 1c). We extended analyses to also assess afferent and efferent regions and associated tracts involved in motor control. Stained area was also significantly lower in SMA grey matter dorsal horn (*P <0.05) and white matter dorsal column (*P <0.05) but did not reach statistical significance in the white matter corticospinal tract (Pns, Supplementary Fig. 1c, g, k).

To assess whether the decrease in total VWF levels were the result of decreased vessel density in SMA, we quantified the number VWF positive vessel profiles by applying design-based stereology. Vessel density was not lower in SMA cord in any of the 4 regions of interest, rather increasing with increased survival time in both ventral horn (Fig. 1d) and dorsal horn (Supplementary Fig. 1d). Indeed, Ki67 labelling revealed that endothelial cells, were frequently proliferating in the SMA spinal cord, but not in controls (Supplementary Fig. 1m-n). For SMA cases that received artificial ventilation during clinical care, no correlation was observed with increased vascular vessel density and endothelial proliferation. In summary, the decrease in total VWF could not be attributed to lower vessel density in SMA.

Release of VWF from endothelial cells is a marker of dysfunction, therefore as the overall reduction in VWF expression was not associated with vessel loss, we next assessed if it was associated with reduced VWF expression per vessel. VWF stained area was reduced by an average of 60% in individual vessel profiles in SMA spinal cord compared to age-matched controls, control = 102μm^2^ (±12) and SMA = 41μm^2^ (±5), mean (±SEM), **P <0.005 (Fig. 1e). Platelets, which also express VWF, were not observed within vessels, and did not affect our assessments.

**Figure 2:**
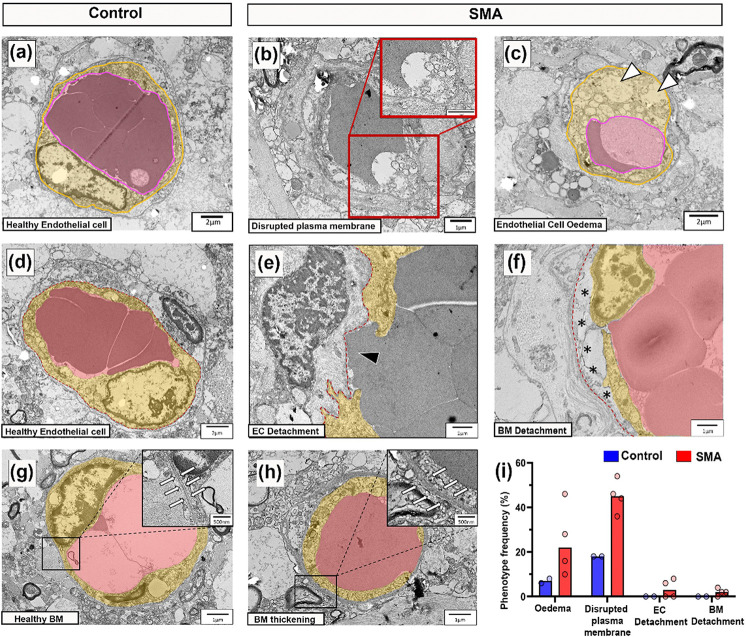
Endothelial integrity is disrupted in the SMA spinal cord (a) Representative electron micrographs of healthy endothelial cell morphology from control spinal cord, scale, 2μm. (b) SMA endothelial cell with disrupted plasma membrane, high power micrographs shown in insert, scale, 1μm (c). SMA endothelial cell with swollen, vacuolated cytoplasm (white arrowheads) indicative of oedema, scale, 2μm. (d) Control healthy endothelial cell, scale=2μm. (e) High power electron micrographs of an SMA vessel with discontinuous endothelial cells forming gaps between adjacent cells, (black arrowhead), scales, 2μm. (f) SMA blood vessel showing detachment of basement membrane from endothelial cells (highlighted by black asterisks), scale, 1μm. (g) Healthy blood vessel from control spinal cord with inset of normal basement membrane (surrounded by white arrows), scale, 1μm. (h) SMA blood vessel with inset of thickened basement membrane (surrounded by white arrows), scale, 1μm. Endothelial lumen (pink) and cytoplasm (yellow) annotated. (i) Frequency of pathological vascular phenotypes observed in control and SMA spinal cord from a total of 50 grey matter vascular profiles evaluated per case. Control n=2 and SMA n=4.

Given our observation of reduced VWF in vessel profiles, we next used transmission electron microscopy (TEM) in a subset of age-matched cases (0–1 year) to determine if reduced VWF per vessel profile in SMA spinal cord was due to changes in the size or number of endothelial cells in each vessel profile. However, no differences were observed between SMA and control cases in endothelial cell area (Fig. 1h–j): control = 38.15μm^2^ (±12.56), SMA = 30.61μm^2^ (±3.54), mean (±SEM), nuclear frequency (as a surrogate for endothelial cell number: Fig. 1l): control range = 0.33–0.55, SMA range = 0.23–0.65 (Fig. 2h), or luminal diameter (Fig. 1j–l): control = 5.69μm^2^ (±0.39), SMA = 5.55μm^2^ (±0.43), mean (±SEM).

These findings show that the decrease in total VWF expression is independent of vessel density, cell number or size, rather representing decreased VWF per endothelial cell in the SMA spinal cord.

### Endothelial integrity is disrupted in the SMA spinal cord

We extended evaluation of endothelial cell health to the ultrastructural level, examining cross sections of vessels in electron micrographs from four SMA and two control cases (demographics in Table 1). Vessels imaged were likely small arterioles, venules and capillaries, as larger vessels are typically associated with the meninges or are travelling in the plane of the section and rarely observed cross-sectionally [65].

In control spinal cords, vascular endothelial cells displayed classical features: flattened nuclei against the vessel wall, electron-dense cytoplasm, and intact luminal membranes [24] (Fig. 2a). In contrast, SMA vascular endothelial cells exhibited disrupted plasma membranes (Fig. 2b), cytoplasmic oedema, organelle disruption, and vacuolisation (Fig. 2c), all indicative of a loss of cellular integrity and compromised cell health. These abnormalities were specific to endothelium, and surrounding glial cells appeared unaffected.

Endothelial cells form the inner layer of the blood spinal cord barrier (BSCB), establishing a continuous monolayer of cells adjacently connected by tight junctions and anchored to the underlying basement membrane on their basal surface [23]. A healthy endothelial cell from control spinal cord (segmented in yellow) with underlying basement membrane (red dashed line) is shown in Fig. 2d for reference. In SMA, endothelial discontinuities were evident in two of four cases, with gaps in the endothelial lining allowing direct blood cell contact with the basement membrane (Fig. 2e). Basement membrane abnormalities, including detachment from the endothelial layer, 3/4 cases, (Fig. 2f) and focal thickening, 4/4 cases, (Fig. 2g–h) were also observed in SMA but not in controls. Endothelial intracellular oedema and membrane rupture were the most consistent pathological features in SMA cases (Fig. 2i).

### Pericyte and Astrocyte End-Feet abnormalities in SMA Spinal Cord

Having identified pathological changes to the endothelial cell layer and basement membrane, we next examined pericytes and astrocyte end-feet, the remaining key cellular components of the BSCB.

**Figure 3:**
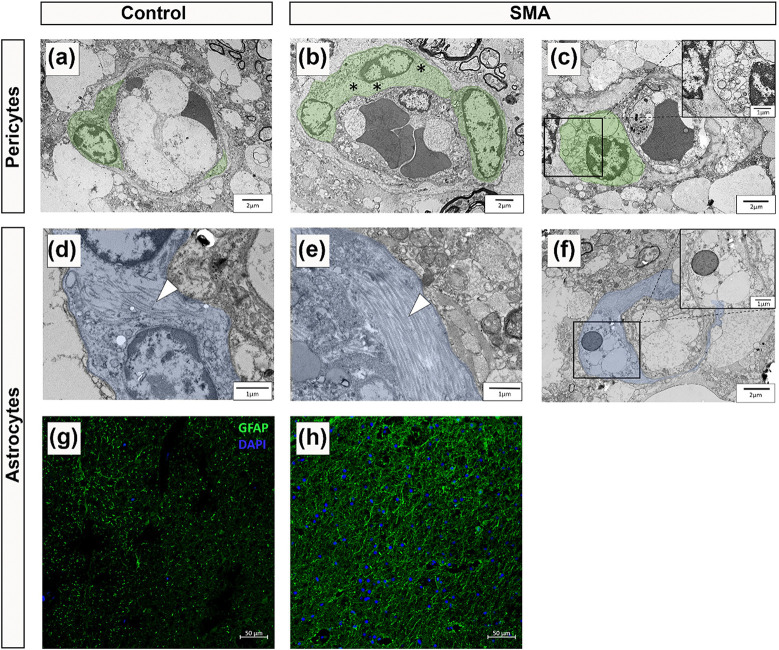
Pericyte and Astrocyte End-Feet abnormalities in SMA Spinal Cord Representative electron micrographs of (a) a healthy pericyte from control spinal cord, scale 2μm. SMA pericytes with (b) large regions of cellular oedema (highlighted by black asterisks), scale 2μm and (c) significant cytoplasmic vacuolisation, scale 2μm, magnified in inset, scale 1μm. (d) A healthy astrocyte from control spinal cord showing few intermediate filaments in the cytoplasm (white arrowhead), scale 1μm. (e) SMA astrocytes with abnormal accumulations of intermediate filaments in the cytoplasm (white arrowhead), scale 1μm and (f) significant cytoplasmic vacuolisation, scale 2μm, magnified in inset, scale 1μm. Astrocytes are segmented in blue. For TEM, control n=2 and SMA n=4. Representative fluorescent micrographs of (g) control and (h) SMA spinal cord stained with glial fibrillary acidic protein (GFAP), an astrocytic marker. SMA spinal cord shows increased expression of GFAP. scale 125μm. For fluorescence microscopy, Control n=5 and SMA n=5.

In control spinal cord, pericytes displayed classic “bump on the log” morphology, lying adjacent to the endothelium with prominent nuclei, limited cytoplasm and long processes wrapping the vessel wall (Fig. 3a) [7]. In contrast, SMA pericytes showed cytoplasmic swelling, oedema (Fig. 3b), and vacuolisation (Fig. 3c).

Astrocyte end-feet form the outer BSCB layer, and appeared normal in control spinal cord with sparse cytoplasmic filaments [9, 45], (Fig. 3d). This contrasts with the astrocytic end-feet visualized in the SMA cases that displayed abnormal filament accumulation (Fig. 3e) and cytoplasmic vacuolisation (Fig. 3f). Immunofluorescence staining showed a visible increase in glial fibrillary acidic protein (GFAP) expression across all spinal cord regions in SMA cases (Fig. 3g–h).

### Evidence of BSCB Compromise in the SMA spinal cord

Given our ultrastructural findings and prior evidence of vascular impairment in both SMA patients and animal models, we next looked for pathological markers of BSCB dysfunction by analysing the distribution of fibrinogen and haemoglobin by immunohistochemistry.

**Figure 4:**
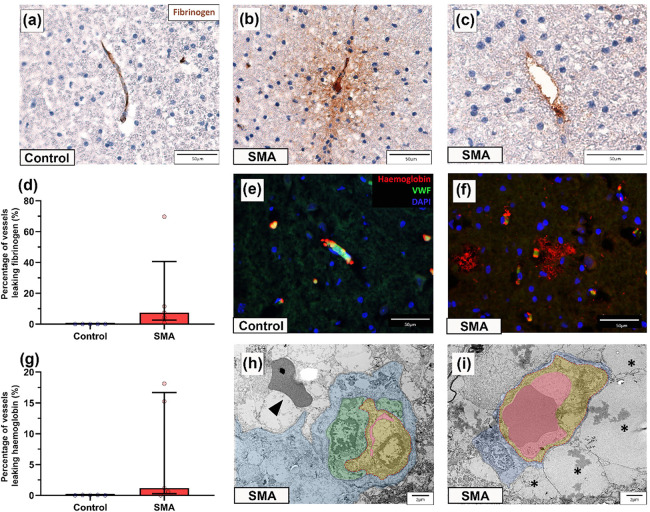
Evidence of BSCB Compromise in the SMA spinal cord (a-c) Representative micrographs of (a) control and (b-c) SMA spinal cord, immunohistochemically labelled with fibrinogen showing (b) diffuse extravascular fibrinogen staining and (c) perivascular fibrinogen deposition, scales 50μm. (d) Percentage of vessels showing extravascular fibrinogen in control and SMA spinal cord. Each value represents the percentage of vessels leaking per spinal cord, median ±interquartile range. (e-f) Representative immunofluorescence micrographs of (e) control and (f) SMA spinal cord labelled for haemoglobin, VWF and DAPI, scales 50μm. (g) Percentage of vessels showing extravascular haemoglobin in control and SMA spinal cord. Each value represents the percentage of vessels leaking per spinal cord, median ±interquartile range. Control n=5 and SMA n=5, 3 sections analysed per case. (h-i)) Representative electron micrograph of an (h) SMA vessel with swollen endothelial cells obstructing the vessel lumen and an extravasated red blood cell (black arrowhead), scale 2μm, and (i) large regions of extracellular oedema surrounding vessels (back asterisks), scale 2μm. In electron micrographs vessel lumens are segmented in pink, endothelial cells in yellow, pericytes in green and astrocytes in blue. For TEM, control n=2 and SMA n=4.

Fibrinogen, a large plasma protein normally restricted to the vascular lumen [51], where it was confined in control spinal cords (Fig. 4a). In contrast, extravascular fibrinogen was detected frequently in SMA spinal cord, presenting as either diffuse staining around the vessel or as focal perivascular deposition (Fig. 4b–c). Extravascular fibrinogen was present in all five SMA cases (median 7. 45%, range 0.95–69.76%) (Fig. 4d), most prominently in the dorsal horn, but not observed in controls (median 0.00%, range 0.00–0.04%).

Haemoglobin, which may be released during or shortly after vascular injury [66], was co-stained with VWF to localise its presence in relation to vessels. In controls, haemoglobin was present intravascularly (Fig. 4e), whereas in SMA spinal cords, extravascular haemoglobin was found surrounding vessels in the neural parenchyma (median 1.17%, range 0.00–18.14%) (Fig. 4f–g). Extravascular haemoglobin was most often seen in the dorsal column.

At the ultrastructural level, extravasated red blood cells were observed in 2 of 4 SMA cases, but not in controls (Fig. 4h), while perivascular oedema was a consistent feature across all SMA spinal cords examined (Fig. 4i).

### Pronounced Microglial Activation is present throughout the SMA spinal cord

**Figure 5:**
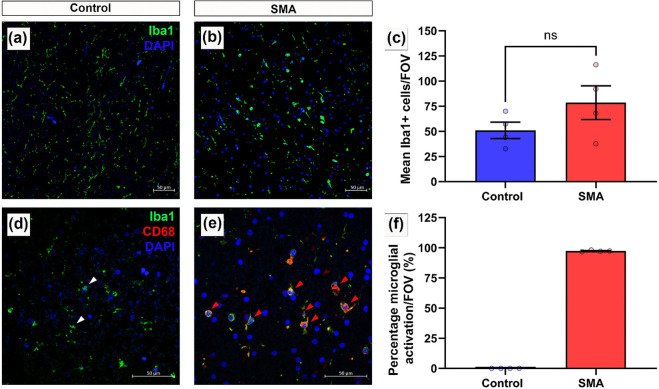
Pronounced Microglial Activation is present throughout the SMA spinal cord (a-b) Representative confocal micrographs of immunofluorescence microglial staining (Iba1 – green) in (a) control spinal cord showing ramified, resting state morphology and (b) in SMA spinal cord, maintaining ameboid morphology indicative of a reactive phenotype. DAPI shown in blue. Scale 50μm. (c) Number of Iba1^+^ microglia observed per FOV in control and SMA spinal cord, mean ±SEM. (Welch’s T test, Pns <0.05). (d-e) Representative confocal micrographs of multiplex immunofluorescence for microglia (Iba1 - green) and macrophage activation marker (CD68 – red) in (d) control and (e) in SMA spinal cord, showing extent of microglial activation. Scale 50μm. White arrowheads indicate resting microglia (Iba1+) and red arrowheads indicate activated microglia (Iba1^+^/CD68^+^). (f) Percentage of microglial activation (Iba1^+^/CD68^+^) in control and SMA spinal cord, mean ±SEM. Control n=4 and SMA n=4.

The presence of plasma proteins in the neural parenchyma suggests vulnerability to BSCB disruption in SMA. A downstream consequence is the potential for reactive neuroinflammation associated with glial activation [67]. This was assessed by microglial Iba1 immunofluorescence in all regions of interest. In control spinal cords, microglia exhibited a ramified morphology typical of a resting state (Fig. 5a). In comparison, SMA microglia were clustered, with an ameboid morphology, retracted processes and reduced branching, consistent with an activated and reactive microglial phenotype [47], (Fig 5b). Microglial cell number was broadly similar in SMA and controls (control: 51 ± 8; SMA: 78 ± 17 Iba1^+^ cells per FOV, Pns <0.05), (Fig. 5c). Microglial activation was increased in SMA cases, where almost all Iba1 positive microglia co-expressed the macrophage activation marker CD68 (97.36% ± 0.29), compared to none in controls (Fig. 5d–f). This reactive microglial phenotype was observed throughout the spinal cord, not only in the ventral horn where motor neuron degeneration occurs, suggesting microglial activation was a widespread characteristic in these cases of SMA.

## Discussion

We have identified abnormalities in SMA spinal cord endothelial cells and BSCB components that are associated with BSCB disruption, leakage and microglial activation. Together these suggest a meaningful, previously underappreciated, role of vascular dysfunction in the pathogenesis of human severe SMA.

### Changes in endothelial VWF identifies endothelial call pathology in SMA spinal cord

VWF is a key endothelial protein essential for maintaining vascular health and haemostasis [40]. We observed reduced endothelial VWF in SMA spinal cords and localized this specifically to individual endothelial cells, reflecting lower intracellular levels within endothelial cells. Changes in intracellular VWF can indicate pathology, as it is rapidly released from endothelial cells upon injury or activation [46]. VWF is normally stored in Weibel–Palade bodies and rapidly secreted into the bloodstream in response to stress or damage—a key event in endothelial activation [5, 37, 38, 50, 55, 59]. In SMA, pathological factors such as hypoxia and oxidative stress—both reported in animal models [29, 63]—could trigger this release [8, 26, 53]. In vitro, hypoxic stimulation of HUVECS increases VWF secretion four-fold [5]. Moreover, superoxide anions generated during hypoxia elevate intracellular Ca^2+^, triggering rapid Weibel–Palade body exocytosis [69]. These reactive oxygen species also promote stress fibre formation and disrupt barrier integrity by disassembling tight and adherens junctions through MAP kinase activation [28, 39]. These mechanisms align with the observations in this study of discontinuities in endothelial vessel walls creating gaps in the barrier, detachment of endothelial cells from the basement membrane, and in previous studies, claudin-5 reorganisation in SMA mouse models [63].

### Basement membrane, pericytes and astrocyte layers of the BSCB exhibit pathological changes

Endothelial cell injury was accompanied by abnormalities in other BSCB components. The basement membrane (BM) in SMA spinal cords was focally thickened, potentially as the result of endothelial dysfunction and serum protein exposure that alter its normal composition [68]. Oxidative stress can disrupt endothelial metabolism, driving abnormal production or turnover of BM components, contributing to its thickening [22]. Similar BM thickening occurs in Alzheimer’s and Parkinson’s disease, where it is thought to be secondary to vascular injury and oxidative stress, and contributes to impairment of vessel contractility, exacerbating local hypoxia [10, 22].

Pericytes in the SMA spinal cord displayed pronounced cytoplasmic swelling, oedema, and vacuolisation, indicative of cellular stress and dysfunction. Given their roles in vessel stability, angiogenesis regulation, and junctional integrity, pericyte impairment could also exacerbate endothelial detachment, disrupt vessel maturation, and compromise barrier function [16, 17].

Astrocytes in the SMA spinal cord showed cytoplasmic vacuolisation and abnormal glial fibrillary acidic protein (GFAP) accumulation. Consistent with our findings, increased GFAP is reported in a separate study of post-mortem spinal cords from severe SMA patients, and SMA mouse models show reactive astrocytes with morphological and functional changes preceding neuronal death [42, 56]. Astrocytes also contribute to the expression of tight junction proteins, including Occludin, Zona Occludens-1, and Claudin-5, which regulate endothelial cell adherence and BSCB integrity [13]. Dysfunction in astrocytes may therefore further contribute to the endothelial cell detachment observed here and the loss of Claudin-5 reported in SMA mouse models [63]. While the phenotypes observed in pericytes and astrocytes may arise as secondary effects of endothelial injury due to complex intercellular communication, they may also reflect intrinsic defects stemming from SMN protein deficiency.

### Pathological changes to the BSCB are associated with barrier dysfunction, leakage and microglial activation

Damage to all components of the BSCB was associated with barrier leakage, demonstrated by the presence of extravascular fibrinogen, a large plasma glycoprotein that enters the neural parenchyma only under pathological conditions [32]. In SMA spinal cords, two patterns of extravascular fibrinogen were observed: diffuse staining surrounding vessels (likely reflecting recent or ongoing leakage) and perivascular deposits (likely remnants of past leakage) [52]. Extravascular fibrinogen drives CNS pathology, triggering microglial activation, promoting neuroinflammation, and inhibiting remyelination and neuronal repair [1, 18, 52, 60].

Extravascular haemoglobin was detected in some SMA cases but did not differ significantly from controls, therefore it may reflect peri-mortem red blood cell lysis or post-mortem artefact. Haemoglobin is known to diffuse following red cell rupture, particularly under conditions of tissue hypoxia or delayed fixation, limiting its utility as a marker of true barrier compromise. The absence of a corresponding increase of extravascular haemoglobin supports our interpretation that fibrinogen leakage reflects true pathological barrier breakdown rather than non-specific post-mortem events.

Increased microglial activation was also present in SMA spinal cords, and extravascular fibrinogen may have contributed to this. When present in the CNS, fibrinogen binds to the integrin receptor Mac-1 (CD11b/CD18) on microglia, triggering intracellular signalling that promotes adhesion, migration, and pro-inflammatory activation [1, 60]. SMA mouse models show reactive microgliosis around motor neurons, characterised by increased M1 and reduced M2 microglial profiles. Initially thought to be secondary to motor neuron degeneration, though more recent work with SMA-derived iPSCs has revealed intrinsic microglial defects in morphology, phagocytosis, and migration [33]. This suggests that microglial activation in SMA might arise from intrinsic pathology and/or extrinsic triggers such as barrier breakdown, creating a sustained pro-inflammatory environment.

### Implications of vascular dysfunction for SMA patients

We have reported extensive pathology in severe SMA patient spinal cords, including all components of the neurovascular unit. Whether these arise secondary to motor neuron loss or are instead a primary consequence of SMN deficiency, this vascular pathology may contribute to a hostile microenvironment for neurons that possibly exacerbate processes of neurodegeneration. While SMN deficiency in motor neurons is a well-established driver of disease, neuron-specific knockdown models do not fully replicate the severity of patient symptoms [48]. Conversely, restoring SMN in peripheral, non-neuronal cells improves survival and motor function, emphasising the contribution of non-neuronal tissues to disease progression [30, 54].

Hypoxia is a critical determinant of neuronal health, and SMN-deficient motor neurons are particularly vulnerable to oxygen deprivation [29]. We propose that endothelial defects in SMA, which could be the result of intrinsic mechanisms through SMN depletion or environmental cues such as hypoxia, drive chronic vascular injury and BSCB dysfunction. This allows toxic blood-derived proteins such as fibrinogen to enter the parenchyma, exacerbating motor neuron degeneration. Even without primary neuronal injury, vascular pathology can create a pro-inflammatory, hypoxic environment that motor neurons are particularly vulnerable to [34].

### Limitations of this study

A well-recognised limitation of autopsy-based studies is variability in premorbid health and cause of death between disease and control groups. In our cohort, control infants died from acute causes such as sudden infant death syndrome (SIDS), asphyxia, or other unknown events, likely without prolonged metabolic compromise (Table 1). In contrast, clinical experience suggests that SMA infants often experience extended periods of respiratory failure, malnutrition, and metabolic stress prior to death.

Notably, PMIs were generally longer in control cases, which may also influence tissue preservation and antigenicity. While such confounds cannot be fully controlled in human autopsy studies, complementary findings from SMA animal models—where vascular changes and BSCB abnormalities are also observed [29, 63]—support the disease relevance of our results. Our findings are presented within this context, and we caution against overinterpretation of differences that could reflect systemic pre-terminal factors.

## Conclusion

Although three disease-modifying therapies now prolong survival in severe SMA it is unknown if any of these will address vascular pathology. Our findings indicate that vascular defects may contribute to motor neuron vulnerability and disease progression. Therefore, long-term monitoring and therapeutic strategies that consider vascular health may improve outcomes of SMA patients receiving these therapies.

## Supplementary Material

Supplementary Files

This is a list of supplementary files associated with this preprint. Click to download.

• floatimage2.png

## Figures and Tables

**Table 1:** Patient demographics Summary of case information including age at death (in days (d) or months (m)), cause of death, SMN2 copy number, post-mortem interval (PMI) where known, and use of samples for light microscopy (LM) and/or transmission electron microscopy (TEM).

	Age of death (days/months)	Cause of death	SMN copy number (*SMN1/SMN2*)	PMI (hours)	Application
**Control Cases**	1d	Unknown	-	25	TEM
	4d	Unknown	-	69	TEM
	9 d	Unknown	-	Unknown	LM
	3.2 m	Sudden unexplained infant death	-	27	LM
	5.2 m	Viral syndrome/focal acute pneumonia	-	31	LM
	7.1 m	Asphyxia	-	24	LM
	9 m	Pneumonia	-	14	LM
	12 m	Sudden infant death syndrome	-	27	LM
**SMA Cases**	7 d	SMA Type 0	Unknown	Unknown	LM
	1.75 m	SMA Type I	0/2	7	LM
					TEM
	2.5 m	SMA Type I	0/2	7	LM
					TEM
	4 m	SMA Type I	0/2	4	LM
	7 m	SMA Type I	0/2	25	LM
					TEM
	11 m	SMA Type I	0/2	32	LM
	11m	SMA Type I	0/2	Unknown	TEM

## Data Availability

The datasets generated during the current study available from the corresponding author on reasonable request.
